# The paradox of efficiency: institutional interfaces, residuals, and the erosion of innovation drivers in AI-augmented organizations

**DOI:** 10.3389/frai.2026.1864238

**Published:** 2026-09-11

**Authors:** Tianyuan Yang

**Affiliations:** 1 College of Management, Hebei GEO University, Shijiazhuang, China; 2 Strategy and Management Base of Mineral Resources in Hebei Province, Hebei GEO University, Shijiazhuang, China

**Keywords:** artificial intelligence, hypothesis and theory, innovation drivers, institutional interface, paradox theory, residuals

## Abstract

As artificial intelligence (AI) becomes deeply embedded in organizational cognition and decision-making, a profound paradox emerges: AI significantly enhances operational efficiency while systematically eroding the generation and retention of innovation drivers. This study introduces the institutional interface as a meso-level analytical lens for examining how specific configurations of AI design choices, human–AI interaction protocols, and organizational norms produce systematic biases. Under the design conditions theorized in this article, these configurations tend to erode innovation drivers through unconscious automatic execution and residual production. This study illuminates a key feature of AI-driven institutional execution—unconscious automatic execution—and deconstructs it into three dimensions: opacity of execution, loss of agency, and diminished reflexivity. It introduces the concept of residuals to capture the cognitive, behavioral, and value-laden elements that deviate from statistical mainstreams or resist quantification and are systematically deprioritized or excluded from organizational awareness. Under specific design conditions and viewed through the three nested interface lenses, AI systems tend to filter residuals across the cognitive, action, and value interfaces, systematically excluding potentially valuable innovations and impoverishing organizational cognitive diversity. This study articulates the conditions under which a self-reinforcing tendency toward efficiency lock-in emerges and theorizes the institutional features—non-optimization zones, outlier pathways, and paradox-balancing roles—that may counteract this drift. Critically, it further reveals the persistent and irreducible dilemmas facing such features (isolation, authority, and evaluation), arguing that they are tensions to be continually managed rather than problems to be solved. Advancing testable theoretical propositions, this article offers new analytical tools and design directions for innovation governance in AI-augmented organizations. It contributes directly to discussions on AI's role in business understanding and managerial decision support, while laying the conceptual foundation for a broader research program on the learning and governance of AI-augmented organizations.

## 1 Introduction

In 2025, a Fortune 500 technology company launched an AI-driven creative evaluation system designed to streamline R&D project screening. Within a year, project approval rates dropped sharply, yet the number of new products achieving superior market performance did not increase—it declined. The system systematically favored proposals that “looked like” historically successful projects while quietly filtering out “fringe ideas” that challenged mainstream assumptions. Those marginal proposals, lacking historical data support, were flagged as “low confidence” and automatically eliminated. Some were later evaluated by human experts and recognized as having breakthrough potential—but by then, they had already vanished from the organization's decision-making stream.^1^

This is not an isolated story. Across sectors, a troubling pattern is emerging: the same AI systems that deliver impressive efficiency gains appear to systematically erode the diversity of ideas, tolerance for uncertainty, and space for trial and error on which innovation depends (Nyenno, 2026; Seeber et al., 2020; Haupt et al., 2024).

This phenomenon is not a technical failure. Paradoxically, it is a consequence of AI's technical success. The algorithmic logic of AI is inherently oriented toward efficiency: it fits historical data, optimizes resource allocation, aligns with mainstream values, and executes tasks in closed loops (Haipeter et al., 2024; Raisch and Krakowski, 2021). In doing so, it identifies and deprioritizes anything that does not conform—elements that fall outside the mainstream distribution, cannot be quantified, or do not directly contribute to measurable outcomes (Kuşci et al., 2026; Rodilosso, 2024). These elements—which are termed residuals—the ideas, behaviors, and perspectives that fall outside the patterns AI systems are designed to prioritize.

This paradox—efficiency's success as innovation's failure—points to a deeper theoretical question: when AI becomes a core institutional force within organizations, how does the relationship between efficiency and innovation change? Is the erosion of innovation drivers an incidental byproduct of AI adoption, or is it a systematically produced tendency arising from specific, identifiable design conditions?

This article argues for the latter position. The focus of this study is on AI-augmented organizations—established firms and institutions in sectors such as manufacturing, finance, healthcare, and retail that increasingly rely on AI systems to enhance or automate core operational and decision-making processes. These are not AI-native technology companies, but traditional organizations whose efficiency-innovation tension is reshaped by the introduction of AI into their institutional fabric. This study focuses on optimization-oriented, discriminative AI systems (e.g., predictive scoring, KPI automation, resource allocation algorithms)—the dominant form of AI deployed in established organizational decision-making contexts. This article advances a series of testable propositions that constitute a novel theoretical framework for understanding how AI-mediated institutional processes systematically erode innovation drivers. This study introduces the concept of the institutional interface as a novel analytical lens to capture the structured arrangements through which AI-mediated processes configure what counts as legitimate cognition, action, and value within organizations. The article's primary contribution is the introduction, definition, and analytical elaboration of this core lens and its associated sub-concepts. No empirical data are presented; rather, a theoretically grounded, testable framework is constructed.

This article directly addresses a pressing managerial reality: how organizations can pursue AI-driven efficiency without eroding long-term innovative capacity. By offering a testable framework of institutional interface design, it contributes to AI governance, digital transformation, and strategic decision-support in business contexts—showing how design choices such as default decision configurations and outlier pathways shape organizational outcomes beyond technical performance.

The article proceeds as follows. First, the theoretical background is reviewed and key gaps are identified, including a systematic comparison with recent parallel research. Second, the conceptual framework is developed, defining the institutional interface as an analytical lens, its modes of operation, its application to specific decision points, and its three nested layers. Third, a series of conditional propositions is presented that articulate the mechanisms through which interface design choices create a tendency toward the erosion of innovation drivers and the conditions under which this drift can be counteracted. Fourth, the persistent dilemmas are theorized that any ambidextrous design must navigate. Finally, theoretical and practical implications are discussed, limitations, a research agenda, and a note on methodological reflexivity.

## 2 The efficiency-innovation tension and the limits of existing frameworks

### 2.1 Exploration-exploitation and the paradox perspective

One of the most influential frameworks in organizational learning is March's (1991) exploration–exploitation dichotomy. March posited that organizations must allocate resources between exploration (searching for new possibilities) and exploitation (utilizing existing knowledge), creating a fundamental tension because the two compete for organizational attention and resources (Clauss et al., 2021; Paredes and Olander Roese, 2024; Vlašić et al., 2024). However, paradox theory (Smith and Lewis, 2011) offers a distinct and, which this article argues is a more appropriate lens for the AI context. Paradox theory posits that exploration and exploitation are not merely competing but interdependent—in the long term, exploitation without exploration leads to rigidity, whereas exploration without exploitation becomes unsustainable (Liu, 2025; Li et al., 2024). More importantly, paradox theory focuses on how organizations respond to this tension: defensive responses (e.g., suppressing contradictions and enforcing consistency) reinforce a single-pole logic, marginalizing opposing elements, whereas dynamic responses (e.g., embracing tensions through differentiation and integration) preserve pluralism (Ahn et al., 2025; Engström et al., 2025; Schrage and Rasche, 2021). For instance, Wang and Long (2025), using a dual-path moderated mediation model grounded in the Job Demands–Resources framework, found that AI technology adoption simultaneously enhances employee innovation through increased felt obligation for constructive change while inhibiting innovation through heightened job insecurity. This ambidextrous effect provides preliminary empirical grounding for the paradox perspective advanced here.

This article adopts paradox theory as its primary lens because, in the AI context, the tension between efficiency and innovation is not merely a resource allocation problem but also an issue of institutional legitimacy—AI reshapes the boundaries of legitimate cognition and action by defining what is considered “reasonable” (Akben and Dong, 2025; Hasan, 2025).

### 2.2 AI as an institutional force

Recent organizational research has increasingly examined the institutional impact of AI. AI is not simply a tool but a force that redefines the boundaries of legitimacy (Faraj et al., 2018; Kellogg et al., 2020; Rahwan et al., 2019). It defines “what is reasonable” through training data, “what is trustworthy” through output thresholds, and “what is acceptable” through alignment mechanisms. In this process, AI systems effectively exercise a form of institutional authority by determining which cognitions and actions are recognized as legitimate and which are excluded (Belenguer, 2022; Liu, 2025; Sun and Xu, 2025). Scoggin (2025) notes that learning algorithms are becoming “constituent elements of organizational cognition,” reshaping knowledge production and cultural norms. In this sense, AI systems function as institutional actors that construct and enforce the rules of organizational life, shaping what counts as legitimate knowledge and action in ways that are often taken for granted (DiMaggio, 1988; Rudko et al., 2024).

While the algorithmic management literature (Kellogg et al., 2020) provides a comprehensive account of how algorithmic systems exercise control through direction, evaluation, and discipline, the institutional interface lens complements this work by examining how such controls operate at specific decision points in real time. Whereas algorithmic management analyzes the architecture of control systems, the institutional interface lens focuses on the micro-level execution of institutional rules—particularly the conditions under which this execution shifts toward unconscious automaticity, rendering the controls themselves difficult to perceive and contest (Fenwick et al., 2024). These perspectives are complementary rather than competing: the institutional interface lens helps explain not what controls are exercised, but why they can become so naturalized as to escape organizational awareness.

### 2.3 The institutional production of innovation drivers and alternative explanations

Innovation drivers—heterogeneous cognitions, boundary exploration, and agency with innovative potential—are recognized, cultivated, or suppressed through specific institutional arrangements (Garud et al., 2018). When institutional processes prioritize certainty and measurability, carriers of uncertainty are systematically produced as residuals (Suchman, 1995). Research on cognitive diversity further demonstrates that heterogeneous cognition constitutes a critical resource for solving complex problems and generating breakthrough innovations (Page, 2007; Vincent et al., 2025; Cohen and Levinthal, 1990).

This study adopts paradox theory (Smith and Lewis, 2011) as its primary analytical lens for re-examining these alternative explanations. Rather than treating market selection, attention constraints, and incentive structures as independent competing theories, the paradox perspective suggests that they may represent surface-level manifestations of a deeper institutional dynamic: the defensive responses through which organizations suppress the efficiency–innovation tension rather than embracing it dynamically. Consequently, the discussion below does not seek to refute these explanations but to specify the institutional conditions under which each operates—and to identify the distinctive predictions that distinguish the institutional interface account from each of them.

The market selection explanation posits that filtered ideas are excluded because they genuinely lack market or technical merit. Although plausible, this explanation assumes that AI filters ideas based on a reliable, preexisting signal of merit (or lack thereof). This framework questions the neutrality of that signal. AI training data and optimization metrics are social and historical constructs rather than transparent reflections of objective market value. The issue is not that AI makes occasional errors—as all selection systems do—but that AI's efficiency logic introduces a systematic and opaque bias against structurally defined residuals, a bias that can become self-reinforcing. In contrast, the institutional interface predicts that exclusion operates prior to evaluation—filtered ideas are not assessed and rejected, but rather rendered structurally invisible. The two accounts thus diverge on whether exclusion is a consequence of assessed merit (market selection) or of pre-evaluative structural filtering (institutional interface).

The organizational attention explanation suggests that AI generates cognitive overload, crowding out attention to marginal ideas. While attention scarcity is undoubtedly a real organizational phenomenon, the institutional interface framework predicts that structural exclusion operates through a distinct mechanism that cannot be reduced to attentional triage. Even when organizational members have spare attentional capacity, filtered residuals may remain invisible because they never enter the decision stream to begin with. The two accounts thus diverge on whether marginal idea invisibility is reversible through attentional redeployment (attention account) or whether it persists regardless of attentional capacity (institutional interface).

The incentive structure explanation argues that employees engage in self-censorship to avoid negative career consequences associated with challenging AI-generated recommendations. This article contends that this explanation, while plausible, captures only one possible pathway—and that the institutional interface account offers a distinct, additional mechanism. The two accounts thus diverge on whether removing external incentives for self-censorship restores idea expression (incentive account) or whether suppression persists due to internalized institutional filtering (institutional interface).

To integrate the preceding discussion, the three alternative explanations can be distinguished along two dimensions: the assumed source of exclusion (whether ideas are actively rejected, cognitively crowded out, or behaviorally suppressed) and the predicted reversibility of the effect (whether removing AI-related constraints restores innovation inputs). The institutional interface framework offers a unified account that (a) identifies the design conditions under which each alternative explanation is most likely to operate, (b) specifies observable predictions that distinguish it from each alternative, and (c) provides a mechanism—structural exclusion through the institutional interface—that the alternative explanations do not capture. This integration positions the framework not as a competitor to existing explanations but as a deeper institutional account that specifies the conditions under which these surface-level dynamics unfold.

### 2.4 Positioning the present framework in the contemporary literature

Several recent studies have examined related paradoxes at the intersection of AI, organizations, and innovation. To clarify the distinctive contribution of the framework, this study offers a direct comparison.

Raisch and Krakowski (2021) laid essential groundwork by identifying the automation–augmentation paradox. This framework builds directly on their insight. This article accepts their core premise that a bias toward automation can lead to capability erosion. The contribution of this study is to specify the meso-level institutional mechanisms (cognitive, action, and value interfaces) through which this erosion of innovation inputs occurs and to identify the specific design conditions (e.g., absent outlier pathways and AI output serving as the default) that translate macro-level tension into micro-level, real-time exclusion. In this sense, this study aims to provide the “missing middle” between their high-level paradox and observable organizational outcomes.

Engström et al. (2025) applied a paradox lens to early AI adoption, identifying temporal and relational tensions. This work complements theirs by shifting the analytical focus from organizational members' experience of tension to the institutional mechanisms that produce and reproduce a tendency toward one pole of the paradox. This framework offers a more parsimonious, mechanism-based account that may explain multiple such paradoxes as surface manifestations of a deeper institutional dynamic.

A complementary perspective is offered by Billi and Labraña (2025), who draw on Social Systems Theory to examine the functional equivalence between AI and human expertise. Their call for hybrid approaches aligns with the advocacy for ambidextrous designs; however, this study further specifies the institutional features required to sustain such hybridity and theorize the dilemmas these features introduce.

In summary, the distinctive contribution of this framework lies in its integration of (a) a meso-level analytical lens bridging macro-institutional logics and micro-algorithmic operations, (b) a focus on design conditions that shift execution toward unconscious automaticity, and (c) a systematic account of the nested mechanisms through which innovation drivers are structurally excluded.

Underlying these contributions is a deeper theoretical claim that distinguishes this framework from prevailing approaches to AI and organization. Much of the existing literature treats the tension between efficiency and innovation as a problem of optimization—one to be solved through better task allocation, improved explainability, or more refined training data. In contrast, this framework suggests that this tension may be constitutive rather than contingent. It arises not from imperfections in AI systems that can be engineered away, but from a fundamental asymmetry: the efficiency logic that makes AI valuable is the same logic that, when institutionalized without countervailing mechanisms, systematically contracts the space within which novel cognitions, behaviors, and values can emerge and gain organizational traction. This constitutive quality suggests that the tension cannot be resolved—only governed through ongoing institutional design and reflexive monitoring.

**Figure 1:**
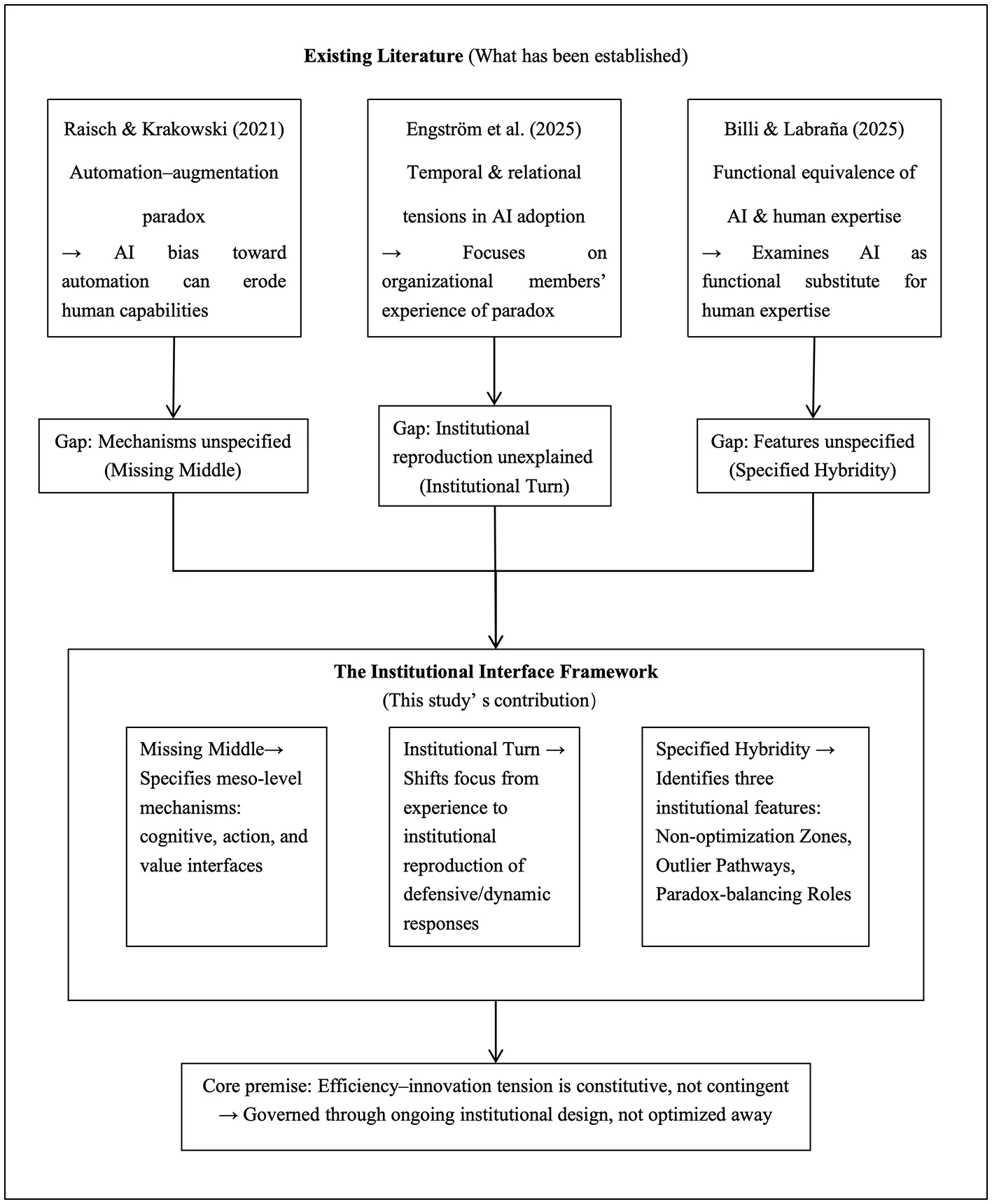
Positioning the institutional interface within the contemporary literature. This figure anchors the framework within the contemporary literature. A systematic conceptual distinction between the institutional interface and adjacent constructs—algorithmic management, governance mechanisms, and technological frames—is provided in Table 2 (Section 3.2).

To crystallize the positioning of the present framework within the contemporary literature, Figure 1 maps the relationship between three streams of existing research and the distinctive contributions offered by the institutional interface lens. As the figure shows, each stream identifies an important aspect of AI's organizational impact—the automation–augmentation paradox (Raisch and Krakowski, 2021), temporal and relational tensions (Engström et al., 2025), and the functional equivalence between AI and human expertise (Billi and Labraña, 2025)—yet each leaves a specific gap that the institutional interface framework is designed to address. The lower portion of the figure synthesizes these contributions into the framework's core premise.

## 3 Conceptual framework: the institutional interface

### 3.1 Defining the institutional interface lens and its modes of operation

This study introduces the institutional interface as an analytical lens for examining how the design, deployment, and routinized use of AI systems within organizations configure an attention structure and a legitimacy filter at specific decision junctures. This lens directs attention to the patterned ways in which certain cognitions, actions, and values gain traction while others fail to register within organizational processes. The institutional interface is therefore not a discrete entity that acts independently of organizational design choices. Rather, it is a perspective that reveals how algorithmic operations, human–AI interaction protocols, and surrounding organizational norms configure a terrain of possibility with systematic biases. The institutional interface is employed as an analytical lens for identifying these configurations, while the propositions theorize their conditional effects (Jaakkola, 2020).

#### 3.1.1 Analytical unit and cross-level variation

The institutional interface is best understood as a practice-level construct. It is instantiated at each specific AI-mediated decision point within an organization, such as a project screening portal, performance evaluation dashboard, or resource allocation algorithm (Orlikowski, 2000). Consequently, a single organization may host multiple institutional interfaces with varying degrees of efficiency bias depending on the specific AI application and its local design parameters. An R&D division's project evaluation AI may operate with different outlier pathway settings than a manufacturing unit's KPI optimization AI (Giddens, 1984). This within-organization variation is not a limitation but a key feature of the framework, as it enables empirical comparison and targeted intervention. Importantly, this lens is applied at the level of specific AI-mediated practices (e.g., an R&D project screening portal or a customer service chatbot interaction).

The distinctive analytical value of the institutional interface lies in its focus on the real-time, automated, and potentially non-reflexive operational layer of institutional execution. To capture this, a distinction is drawn between two ideal-typical modes of operation positioned along a continuum:

Institutional rules are executed by human actors who remain aware of their judgments, can question their criteria, and can deliberately make exceptions. An R&D manager screening proposals who consciously evaluates submissions and decides to retain a marginal idea that “doesn't fit the criteria” but “intuitively has potential” exemplifies reflective compliance.

#### 3.1.2 Unconscious automatic execution

Institutional rules are executed by algorithms that lack awareness of their judgments, do not question their criteria, and make exceptions only when explicitly designed to do so. An AI system that automatically filters “out-of-distribution” proposals based on a confidence threshold—preventing filtered proposals from ever reaching human awareness—represents the extreme form of unconscious automatic execution.

Deconstructing “Unconscious Automatic Execution.” This extreme mode is further deconstructed into three dimensions. The first dimension, Opacity of Execution, captures what earlier formulations might have described as cognitive unconsciousness—not the algorithm's lack of self-awareness, but the human actor's inability to perceive or understand the algorithmic judgment process. Filtering occurs within a “black box,” rendering decision criteria invisible to those affected (Gaddapuri, 2025; Pasupuleti, 2025). The second dimension, Loss of Agency, refers to the difficulty or impossibility for human actors to intervene in or override algorithmic decisions. The third dimension, Diminished Reflexivity, denotes the absence of mechanisms for questioning, revising, or recalibrating the institutional standards encoded in the algorithm over time.

Importantly, AI-driven execution does not inherently occupy the unconscious automatic extreme. Rather, placement along this continuum depends on organizational design choices—for example, whether AI output functions as a default decision or as advisory input. This framework therefore focuses on the conditions under which drift toward the unconscious automatic pole occurs. Table 1 summarizes the dimensions of unconscious automatic execution and the conditions under which it becomes entrenched.

**Table 1:** Dimensions of “Unconscious Automatic Execution.”

Dimension	Core meaning	Traditional institutional execution	AI-driven institutional execution	Conditions for occurrence
Opacity of execution	Whether the executor is “aware” of executing the institution	Conscious: Manager knows they are screening proposals	Unconscious: Algorithm lacks self-awareness	AI system is a “black box” with no explanatory mechanisms
Loss of agency	Whether actors can intervene in the execution process	Intervenable: Manager can retain marginal proposals	Difficult to intervene: Filtered proposals are invisible	System lacks human review pathways
Diminished reflexivity	Whether institutional standards can be questioned and adjusted	Reflexive: Manager can question screening criteria	Non-reflexive: Confidence threshold runs invariantly	System lacks periodic standard review mechanisms

The drift toward unconscious automatic execution, while systematic, is not deterministic. A countervailing force operates when organizational members possess high AI literacy, formal override rights, or institutionalized dissent channels. Under such conditions, actors can consciously interrupt algorithmic judgments, question their criteria, and advocate for excluded alternatives—shifting the interface back toward the reflective compliance pole. This human reflexivity, drawing on the literature on algorithm aversion (Dietvorst et al., 2015) and behavioral responses to algorithmic systems, constitutes an implicit check on the three dimensions of unconsciousness specified above. The specific mechanisms through which actors resist, reinterpret, override, or adapt—and the boundary conditions of these responses—are elaborated in the following propositions and the ambidextrous interface design.

Throughout this article, the institutional interface is employed consistently as an analytical lens. When propositions about its effects are advanced, they theorize how specific configurations of AI design and organizational practice—viewed through this lens—tend to produce particular outcomes, rather than attributing causal agency to the interface itself.

### 3.2 Distinguishing the institutional interface from related concepts

To clarify its conceptual boundaries, Table 2 compares the institutional interface with related concepts, including institutional logics (Thornton et al., 2012), technological frames, and legitimacy boundaries.

**Table 2:** Comparison of the institutional interface with related concepts.

Concept	Core focus	Explanation of AI	Distinctive analytical dimension of the institutional interface
Algorithmic management (Kellogg et al., 2020)	How algorithms control and direct workers through direction, evaluation, and discipline	AI as a tool for managerial control and workplace surveillance	Unlike algorithmic management's focus on the architecture of control and power relations, the institutional interface focuses on the real-time execution layer—how institutional rules are enacted at specific AI-human decision points, regardless of who controls the system.
Governance mechanisms (Mäntymäki et al., 2022)	Rules, practices, and processes that direct and control organizational behavior	AI as an object to be governed through policies and compliance frameworks	Unlike macro-level governance, which operates at the policy and compliance level, the institutional interface operates at the practice level of specific AI-mediated decision points—examining how rules are executed, not how they are formulated or monitored.
Technological frames (Orlikowski, 1994)	People's assumptions, expectations, and knowledge about technology and its use	Meaning of AI is socially constructed by users through interpretation and sensemaking	Unlike technological frames, which center on human interpretive construction of technology, the institutional interface centers on the automated, non-reflexive operationalization of institutional standards—examining how AI executes rules without human awareness or deliberation.

### 3.3 The three layers of the institutional interface

The institutional interface operates across three nested layers. Each layer reflects a mode of operation positioned along the continuum from reflective compliance to unconscious automatic execution.

#### 3.3.1 Cognitive interface

The cognitive interface determines which forms of knowledge, assumptions, and problem definitions enter organizational decision premises. Under reflective compliance, a manager actively seeks marginal ideas and may advocate for their inclusion. Under unconscious automatic execution, algorithms automatically filter “out-of-distribution” inputs based on confidence thresholds, eliminating low-confidence proposals before they reach human awareness.

#### 3.3.2 Action interface

The action interface determines which behaviors are permitted, rewarded, or inhibited. Under reflective compliance, a manager evaluates the value of trial-and-error behavior and may shield exploratory activities from short-term performance pressures. Under unconscious automatic execution, automated KPI systems track behavior in real time, flag activities deemed inefficient, and automatically reallocate resources away from them.

#### 3.3.3 Value interface

The value interface determines which subjectivities and evaluative perspectives are granted authority. Under reflective compliance, scholarly, or professional communities debate research value and defend unorthodox ideas. Under unconscious automatic execution, value-alignment algorithms aggregate mainstream preferences, causing dissenting views to struggle to secure resources and gradually lose institutional authority.

Although analytically distinct, these three layers are nested: the value interface often operates as the deepest layer, with marginalized subjectivities reducing advocacy for outlier review at the cognitive interface, which in turn narrows behaviors at the action interface. This suggests a general sequence—value marginalization preceding cognitive narrowing preceding behavioral constraint—though the coupling is not mechanically tight. In decision-support AI contexts, the cognitive interface may dominate; where AI automates resource allocation, the action interface may be the primary bias site. Table 3 summarizes the characteristics of the three institutional interface layers.

**Table 3:** The three layers of the institutional interface.

Layer	Core function	AI intermediary mechanism	Traditional execution	AI execution (unconscious automatic)
Cognitive interface	Determines which knowledge, assumptions, and problem definitions enter decision premises	Training data distribution, model confidence thresholds, output format presets	Manager judges which proposals are “worth looking at,” can actively retain marginal ideas	System automatically filters “out-of-distribution” proposals, invisible to humans (Yang et al., 2022)
Action interface	Determines which behaviors are permitted, rewarded, or inhibited	Automated KPI evaluation, real-time feedback loops, mandatory workflow paths	Manager evaluates value of trial-and-error behavior, can explain its long-term significance	System flags “inefficient behaviors” in real-time, automatically reallocates resources (Huang and Gursoy, 2024)
Value interface	Determines which subjectivities are granted authority	Value alignment algorithms, preference learning mechanisms, reward/punishment signaling	Academic community debates research value, can defend “unorthodox” ideas	System automatically prefers mainstream paradigms, dissenting views fail to secure resources (Engel et al., 2022)

### 3.4 Residuals: the institutional fate of innovation drivers

Within this framework, residuals refer to cognitive, behavioral, and value-laden inputs that exhibit any of three attributes—statistical rarity, non-quantifiability, or value heterodoxy—and are therefore systematically excluded by AI systems optimized for efficiency. These attributes need not co-occur; an element qualifies as a residual if it possesses even one of them. This reflects the observation that AI efficiency logic can filter inputs through multiple independent pathways, and that the three attributes, while distinct, may carry different organizational implications. Crucially, this definition makes no a priori claim regarding the inherent value or innovation potential of any particular residual. Making such a claim would reproduce the very hubris this framework critiques. Instead, this article argues that the systematic exclusion of structurally defined residuals increases an organization's long-term exposure to errors of incorrectly rejecting potentially valuable ideas and, more importantly, impoverishes the cognitive and normative diversity from which novel recombinations may emerge. The loss is therefore one of potentiality and learning opportunity rather than the disappearance of a predefined set of “hidden gems.”

Importantly, residuals are not intrinsic properties of ideas, behaviors, or perspectives; they are relationally produced through the operation of the institutional interface. An idea becomes a residual only in relation to a specific AI system's training distribution, optimization metric, and decision threshold. The same idea may be mainstream in one organizational unit yet classified as residual in another.

To distinguish residuals from related concepts in the innovation literature, their analytical scope needs to be clarified. Cognitive diversity (Page, 2007) refers to variation in problem-solving approaches within a group, whereas residuals denote specific elements that are actively filtered out by institutional mechanisms rather than simply reflecting a lack of diversity. Redundancy (March, 1991) refers to overlapping resources or behaviors that enhance organizational robustness; residuals may include forms of redundancy but also encompass cognitive and subjective elements that are not redundant yet nevertheless deviate from dominant patterns. Similarly, marginal ideas in creativity research generally refer to novel but potentially valuable suggestions, while residuals are defined by their institutional treatment—they are the ideas, behaviors, or perspectives excluded by the interface regardless of their potential value. The concept of residuals thus shifts analytical attention away from the intrinsic qualities of ideas and toward the institutional processes through which exclusion is produced.

The boundary between residuals and innovation drivers is not fixed. Today's residuals may become tomorrow's paradigm. However, when the institutional interface remains persistently biased toward efficiency, the pathway through which residuals evolve into innovation drivers becomes systematically obstructed.

## 4 Theoretical model: interface bias and the paradox dynamics of innovation driver loss

### 4.1 From algorithmic tendency to institutional bias

This section advances a series of propositions that are both theoretically grounded and empirically testable. Each proposition identifies specific, observable conditions under which the hypothesized mechanisms should operate.

#### 4.1.1 Proposition 1a (algorithmic tendency)

To the extent that AI systems are trained to optimize for measurable outcomes, fit dominant patterns in historical data, and align outputs with majority preferences, they will exhibit a systematic bias toward the efficiency pole—operationalized as a statistically significant preference for mainstream over marginal inputs in their output distributions.

Notably, this algorithmic tendency is expected to be most pronounced in discriminative, high-stakes AI applications (e.g., predictive scoring, resource allocation). Generative AI may partially counterbalance it by expanding combinatorial possibilities, yet may also paradoxically intensify filtering due to hallucination risks and subsequent stricter human-imposed safeguards. Exploratory AI systems, by design, likely attenuate the proposed effects. The propositions below should therefore be understood as context-dependent, with strongest applicability to evaluative, discriminative AI applications in organizational decision-making.

#### 4.1.2 Proposition 1b (design conditions for bias entrenchment)

This algorithmic tendency translates into institutional interface bias when two organizational design conditions are present: (i) outlier pathways are absent, such that low-confidence outputs do not enter human review, and (ii) AI output functions as the default decision, defined as a binary configuration in which AI-generated recommendations are implemented without requiring human ratification as a procedural precondition. This is distinguished from an advisory configuration, in which explicit human approval is procedurally required.

#### 4.1.3 Proposition 1c (mechanisms of entrenchment)

When the conditions specified in Proposition 1b are met, the configuration of AI-mediated decision processes shifts toward the extreme of unconscious automatic execution, and bias becomes entrenched through three sub-mechanisms: (i) Within the cognitive interface, low-confidence or marginal cognitions are automatically filtered and rendered institutionally invisible. (ii) Within the action interface, AI-generated decisions directly trigger resource reallocation without meaningful human intervention. (iii) Within the value interface, algorithmic preference judgments are automatically translated into bases for authority allocation.

#### 4.1.4 Causal mechanism

When outlier pathways are absent, marginal cognitions fail to reach human awareness, effectively closing the cognitive interface. When AI output operates as the default decision, human actors face significant barriers to intervention, compressing reflective space within both the action and value interfaces (Lee et al., 2025). Conversely, when outlier pathways exist and AI output serves an advisory rather than determinative role, human actors retain awareness, agency, and reflexivity. Under these conditions, algorithmic tendencies can be identified, questioned, and adjusted, thereby potentially preventing their translation into institutional bias (Hanna et al., 2024).

Having specified the conditions under which algorithmic tendency translates into institutional bias, the following section examines how this bias, once entrenched, systematically produces residuals through three distinct but interrelated mechanisms.

### 4.2 Three mechanisms for the production of residuals

Empirical research on AI-mediated organizing has documented patterns consistent with the three mechanisms that follow. The discussion below synthesizes these observations into a theoretical account of residual production, while formal testing of the mechanisms is left to future research.

#### 4.2.1 Cognitive mechanism (ghosting effect)

When an AI system's confidence threshold prioritizes the exclusion of out-of-distribution inputs and excluded inputs do not enter human review because an outlier pathway is absent, marginal cognitions are removed from organizational decision premises. Organizational members therefore do not merely reject these ideas—they never encounter them. Because AI training data are derived from historically successful cases, they already reflect earlier filtering processes that excluded heterogeneous pathways that may have failed historically yet could succeed under future conditions. As a result, the AI system becomes structurally incapable of recognizing the breakthrough potential embedded in marginal cognitions (Ziakkas et al., 2025). Empirically, this ghosting effect has been observed in R&D settings where AI confidence thresholds eliminate early-stage speculative designs before human scrutiny (Seeber et al., 2020).

#### 4.2.2 Action mechanism (squeeze effect)

When AI-driven KPI systems prioritize quantifiable and short-term visible behaviors and these evaluations automatically trigger resource reallocation because AI output functions as the default decision, redundant experimentation and trial-and-error behaviors are gradually squeezed out of organizational resource allocation processes. The “inefficient freedom” required for innovation rarely produces measurable short-term outcomes, and optimization logics therefore redirect resources toward more predictable and immediately productive activities (Corsini et al., 2025; Taher et al., 2024). Empirical research on KPI-driven automation has documented this squeeze effect, as real-time performance tracking progressively redirects resources away from exploratory activities that resist short-term quantification (Huang and Gursoy, 2024).

#### 4.2.3 Value mechanism (marginalization effect)

When AI value-alignment mechanisms prioritize conformity to mainstream preferences—typically through the aggregation of extensive historical data—and alignment scores directly influence authority or resource access, heterogeneous subjectivities lose institutional standing. Individuals who maintain counter-consensus perspectives become marginalized because alignment algorithms statistically privilege majority preferences, whereas innovation frequently depends on sustained commitment to minority viewpoints that diverge from prevailing consensus in AI (Spanuth and Urbano, 2023; Suchman, 1995). Studies on value-alignment mechanisms suggest that minority viewpoints systematically receive lower resource priority as aggregation algorithms privilege majority patterns (Purves and Davis, 2022).

These three mechanisms operate not independently but as mutually reinforcing processes. Cognitive ghosting at the cognitive interface reduces the range of alternative behaviors capable of challenging KPI-driven pressures at the action interface. At the same time, the marginalization of value-heterodox actors at the value interface weakens the organizational constituency most likely to advocate for outlier review pathways, thereby reinforcing initial cognitive filtering. This recursive coupling suggests that interventions targeting only one mechanism are unlikely to reverse the broader drift toward efficiency dominance.

### 4.3 Vicious cycle: self-reinforcing defensive paradox responses

The three mechanisms described above do not operate in isolation. When simultaneously active, they reinforce one another in ways that generate a self-reinforcing dynamic, trapping the organization in a defensive posture that becomes increasingly difficult to escape.

#### 4.3.1 Proposition 2a (data feedback)

When AI systems undergo continuous retraining on internally generated data and the data pipeline systematically excludes residuals filtered at earlier stages, training data become progressively more homogeneous across successive cycles, reinforcing the system's preference for mainstream patterns. This feedback attenuates when models remain static or are trained on external datasets (Morandini et al., 2023).

#### 4.3.2 Proposition 2b (cognitive feedback with moderating conditions)

Persistent exposure to homogeneous AI outputs diminishes organizational members' capacity for critical reflection on AI judgments. This effect is moderated by the organizational context: an explicit mandate encouraging members to “expect the unexpected,” combined with sufficient cognitive slack for anomaly examination, may counteract habituation through the oddball effect (Itti and Koch, 2001). In efficiency-maximizing environments lacking such countervailing supports, habituation dominates (Jiang et al., 2025; Parunak et al., 2008).

#### 4.3.3 Proposition 2c (ongoing institutional reproduction)

When actors holding heterogeneous cognitions are marginalized or exit the organization, the internal political capacity to challenge interface configurations weakens, producing an endogenous drift toward institutional inertia.

#### 4.3.4 Proposition 2d (crisis-driven punctuation)

External shocks—market decline, the emergence of a disruptive competitor, or public controversy arising from algorithmic bias—can reopen institutional debate, creating space for previously marginalized ideas and enabling punctuated shifts in interface design.

### 4.4 The ambidextrous interface: from defensive to dynamic responses

If the vicious cycle described in Proposition 2a−2d represents the defensive pole of organizational response, what institutional features might enable a shift toward the dynamic pole? Three such features are now theorized, which collectively constitute the ambidextrous interface.

#### 4.4.1 Proposition 3

When organizations embed specific observable institutional features, these features can interrupt or mitigate the three residual-producing mechanisms and the associated vicious cycle, thereby shifting organizational responses from defensive to dynamic forms.

These three features are theorized as having primarily additive effects: each independently blocks its corresponding mechanism, and the presence of additional features should incrementally strengthen the organization's capacity to shift toward dynamic responses.

(a) Non-optimization zones: Clearly defined domains characterized by minimal AI involvement (e.g., free exploration time or early-stage creative projects not subject to evaluation), preserving the generative conditions necessary for innovation drivers. This zone is characterized by a blocking mechanism. This feature blocks the action mechanism by providing behavioral space free from short-term KPI evaluation, allowing redundancy and trial-and-error learning to survive. The following questions are considered during operationalization: Does the organization have explicitly defined time periods or spatial areas with “low AI involvement”? How are the boundaries of these zones defined?

Consider the research and analysis department of a Chinese securities firm, where a shared knowledge base allows employees to document AI blind spots without prior approval. The knowledge base is reviewed bi-weekly by the product team, which holds authority to determine which observations warrant follow-up investigation. The organization allocates protected, non-KPI working time—such as a half-day weekly exploration slot—and a modest discretionary budget for these activities. An analyst's observation that “AI misses business transition signals” was subsequently used to refine prompt logic—illustrating a non-optimization zone in practice: a protected space where trial-and-error learning is preserved from short-term KPI pressure.

(b) Outlier pathways: These are deliberately designed mechanisms within AI systems that retain low-confidence outputs and route them into structured human review processes. Outlier pathways prevent premature closure of the cognitive interface. A blocking mechanism blocks the cognitive mechanism by ensuring that marginal cognitions reach human decision makers rather than being filtered out at the AI stage. The following questions are considered during operationalization: Does the AI system maintain a separate list of “low-confidence outputs”? Are there periodic human review processes? Are there documented cases of AI judgments being reversed?

In the corporate credit department of a Chinese commercial bank, loan applications flagged as high-risk by the AI are not automatically rejected. Instead, a conditional pathway enables relationship managers with extended client history to initiate a deep-dive review incorporating qualitative data. A monthly boundary review meeting, chaired by the credit director, determines whether a divergence stems from model limitations or gaps in human judgment. The deep-dive process is supported by a dedicated innovation buffer fund that covers the additional costs of these outlier reviews—anchoring the outlier pathway mechanism by which the cognitive interface remains open to structurally excluded inputs.

(c) Paradox-balancing role: Organizational roles specifically designated to maintain the balance between efficiency and innovation, endowed with cross-interface authority to prevent domination by a single logic (Zhang et al., 2015). A blocking mechanism blocks the value mechanism by providing an institutionalized role capable of advocating for heterogeneous subjectivity, preventing mainstream preferences from completely suppressing minority voices. The following questions are considered during operationalization: Is there a clearly defined “paradox management” responsibility? Does this role control resources in both efficiency and innovation domains? Does their performance evaluation include ambidexterity metrics?

In the research production department of a Chinese securities firm, a product manager designed a tiered AI integration protocol. For standardized reports, the AI functions as the primary author under the efficiency logic; for strategic reports, the AI serves only as a research associate, with the senior analyst retaining final responsibility under the innovation logic. The product manager holds cross-functional authority to adjudicate deviations from the protocol and controls a discretionary budget to fund boundary cases. The protocol is reviewed quarterly, and analysts must document instances where their judgment diverges from AI outputs—illustrating the paradox-balancing role as an institutionalized mechanism that preserves human reflexivity at the value interface.

Together, these three features constitute what is termed the ambidextrous interface. Their purpose is not to eliminate the tension between efficiency and innovation but to preserve the institutional legitimacy of innovation, enabling the organization to accommodate both interdependent demands (Zhang et al., 2025). Critically, the effectiveness of these ambidextrous features presupposes the organizational conditions identified above—including members' capacity for critical reflection, the presence of formal override rights, and institutionalized dissent channels. Without these cognitive and institutional preconditions, even well-designed interfaces may be rendered symbolic rather than effective counterweights to efficiency lock-in. The transition from defensive to dynamic responses occurs when organizations move away from suppressing the efficiency–innovation tension and instead consciously design institutional arrangements capable of accommodating both interdependent logics simultaneously.

### 4.5 Inherent paradoxes of the ambidextrous interface

However, the ambidextrous interface itself faces profound inherent paradoxes. Any attempt to “solve” a paradox may generate new tensions. This section identifies and explores three major paradoxes that the ambidextrous interface may encounter:

#### 4.5.1 The isolation paradox

When organizations adopt spatial separation strategies (e.g., innovation labs) to protect innovation drivers, the insulated innovation space may become disconnected from mainstream operations, preventing the translation or implementation of innovations. Boundary explorations within the lab risk becoming decorative additions irrelevant to the organizational core rather than engines of organizational change. Causal mechanism: Isolation protects innovation drivers from efficiency logic but simultaneously blocks interaction with mainstream operations, hindering resource acquisition and organizational influence (Lewis et al., 2019). Mitigation: While maintaining spatial separation, organizations should institutionalize pathways for translating innovations into mainstream operations, such as technology transfer funds, innovation ambassador rotation programs, and periodic innovation showcases.

#### 4.5.2 The authority paradox

Granting paradox-balancing roles cross-interface authority may still result in their capture by either efficiency or innovation logic. For example, if a paradox-balancing role's performance evaluation is primarily determined by short-term efficiency metrics, their behavior will likely favor the efficiency pole despite their nominal balancing role (Batool et al., 2023). Causal mechanism: Granting authority does not guarantee independent exercise of that authority; leaders' behavior is shaped by performance evaluations, organizational expectations, and peer pressures.

#### 4.5.3 Mitigation

Evaluation metrics for paradox-balancing roles should themselves be ambidextrous (including both efficiency and innovation indicators). Their tenure should be sufficiently long to withstand short-term performance pressures, and they should report directly to top management.

#### 4.5.4 The evaluation paradox

Once evaluation of innovation drivers is incorporated into an AI system, it risks being recaptured by efficiency logic (Saxena and Totaro, 2024). For instance, an organization may attempt to use AI to evaluate innovation potential, yet AI evaluation criteria—based on statistical fitting to historical data—are structurally limited in recognizing true breakthrough innovations. Causal mechanism: Any quantifiable evaluation metric becomes an optimization target (Goodhart's Law). When innovation potential is quantified, organizational members may optimize the metric rather than pursue genuine innovation. A subtler manifestation arises from the very visibility of ambidextrous features: when an organization publicly commits to outlier review, the commitment itself may incentivize actors to frame proposals as “overlooked outliers” to secure protected resources, recreating the gaming problem at the level of organizational discourse. Mitigation: Evaluation of innovation drivers should maintain a human-led, AI-assisted structure. Evaluation criteria should be periodically reviewed and adjusted by human experts, and decision-making space should be preserved for considerations that are unquantifiable yet intuitively valuable.

#### 4.5.5 Proposition 4 (theoretical expansion)

While the ambidextrous interface blocks defensive responses, it simultaneously generates new paradoxical tensions (isolation paradox, authority paradox, evaluation paradox). Whether an organization achieves genuine dynamic responses depends on its capacity to recognize and effectively address these secondary paradoxes. Paradox management should therefore be understood as an ongoing process rather than a one-time solution.

**Figure 2:**
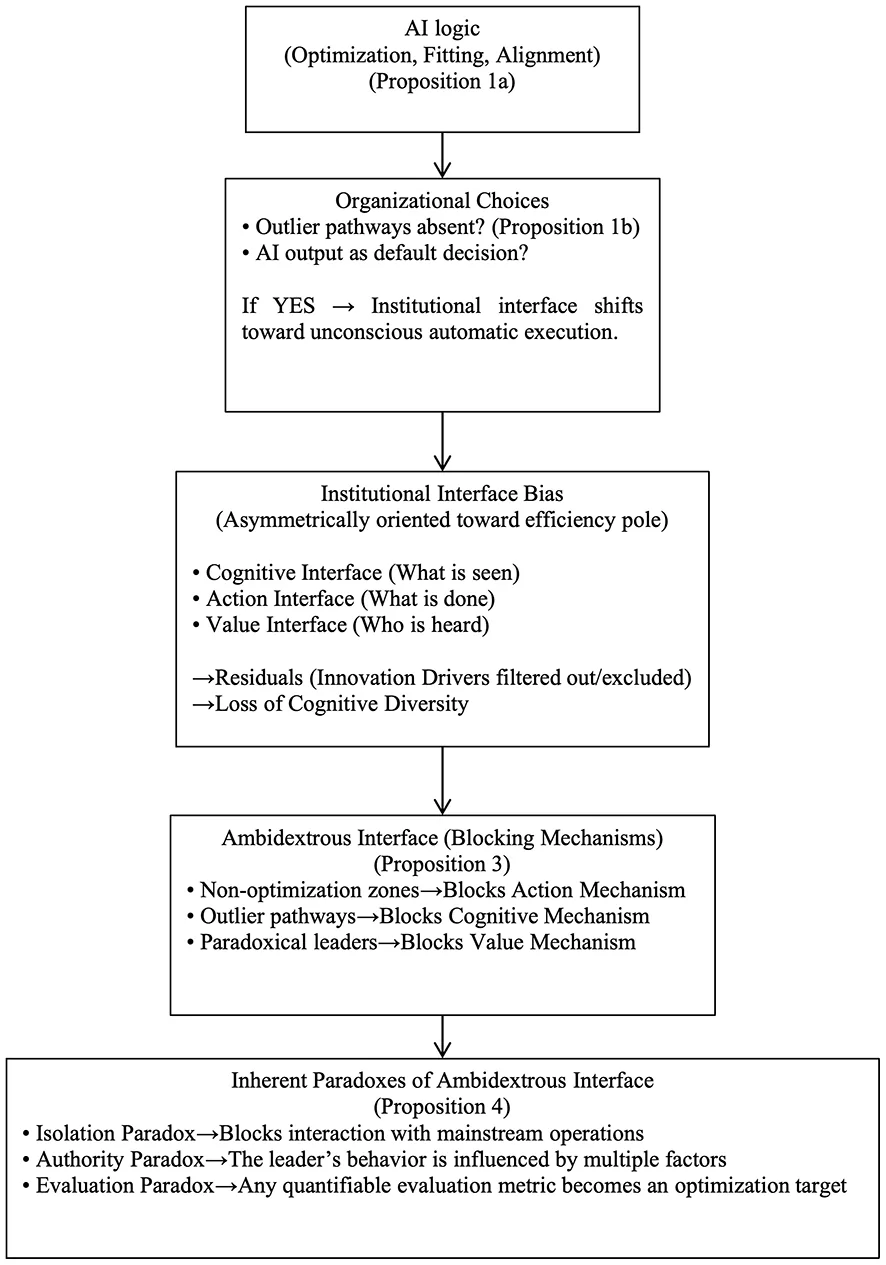
Theoretical model: institutional interface bias and the vicious cycle of innovation driver loss.

Figure 2 below summarizes the complete theoretical model, including the transition from defensive to dynamic responses.

Having articulated the core propositions and the secondary paradoxes they entail, the discussion now turns to the broader implications of the framework for theory and practice, and to the methodological strategies through which these propositions can be subjected to empirical scrutiny.

## 5 Discussion: theoretical contributions, practical implications, and a research agenda

### 5.1 Theoretical contributions

This study makes several theoretical contributions. First, it introduces the institutional interface as a practice-level analytical construct, clarifying its distinction from algorithmic management and its role in naturalizing efficiency logic within organizational cognition. By specifying its analytical unit (AI-mediated decision points) and allowing for within-organization variation, the framework achieves both conceptual precision and empirical tractability.

Second, it refines the concept of unconscious automatic execution by replacing the ambiguous notion of cognitive unconsciousness with Opacity of Execution, a dimension that more accurately captures human actors' inability to perceive algorithmic judgment processes. This refinement, combined with the specification of Loss of Agency and Diminished Reflexivity, provides a more rigorous vocabulary for analyzing the institutional effects of AI.

Third, the analysis offers an ex ante definition of residuals grounded in structural attributes—statistical rarity, non-quantifiability, and value heterodoxy—thereby avoiding the *post hoc* circularity that characterizes many discussions of innovation filtering. The probabilistic framing of residuals as a risk-bearing category acknowledges that not all excluded elements are valuable while preserving analytical attention to the systematic nature of exclusion.

Fourth, it develops conditional propositions for the ambidextrous interface, recognizing that features such as non-optimization zones and outlier pathways are effective only when combined with specific enabling conditions, including translation pathways, counter-lock-in review structures, and ambidextrous evaluation systems. This shifts the discussion beyond prescriptive checklists toward a more nuanced theory of organizational design.

Fifth, the framework integrates organizational reflexivity and the challenges of escaping vicious cycles, linking the analysis to broader debates on organizational learning and adaptive capacity in AI-intensive environments.

### 5.2 Practical implications

The framework carries direct implications for AI system design in business contexts. Organizations should recognize that the institutional interface—the real-time execution of institutional rules at AI-human decision points—actively shapes what counts as legitimate cognition, action, and value. The principles below translate theoretical propositions into actionable guidance organized around three objectives: preserving cognitive diversity, maintaining human reflexivity, and preventing efficiency logic from becoming self-reinforcing.

Establish non-optimization zones with translation pathways. Organizations should define domains with minimal AI involvement—such as free exploration time or early-stage projects not subject to AI evaluation—to preserve generative space for innovation drivers. Crucially, these zones must be coupled with institutionalized translation mechanisms, including innovation funds, ambassador rotation programs, or periodic innovation showcases, that enable ideas to migrate into mainstream operations. Without such pathways, protected spaces risk becoming symbolic rather than transformative.

Design outlier review processes that counteract cognitive lock-in. AI systems should be engineered to retain low-confidence outputs for structured human review. Effective practices include cognitively diverse review panels, blind evaluation procedures, and explicit training aimed at recognizing cognitive heterogeneity. Generating an “outlier list” without meaningful institutional attention does not prevent interface lock-in.

Create paradox-balancing roles with ambidextrous evaluation. Organizations should establish leadership roles explicitly responsible for maintaining balance between efficiency and innovation. These roles require evaluation systems incorporating both efficiency and innovation indicators, sufficiently long tenure to resist short-term performance pressure, and direct reporting relationships with top management to safeguard independence and cross-interface authority.

Maintain independent signal channels. Organizations should preserve mechanisms that deliberately bypass the institutional interface—anonymous innovation suggestion systems, internal dissent channels, or periodic shadow reviews conducted without access to AI-generated scores. By comparing outputs from these channels with formal AI-mediated decisions, organizations can detect systematic exclusion patterns before cognitive lock-in renders such self-correction impossible.

Cultivate executive paradox mindset. Senior leaders need to develop the cognitive capacity to tolerate and actively manage contradictory demands—a capacity that can be cultivated through formal training interventions that expose leaders to paradoxical decision scenarios, embedding paradox navigation metrics into leadership performance evaluations, and incorporating paradox mindset assessment into executive selection criteria (Marvi et al., 2025). A paradox mindset encourages leaders to intentionally seek, protect, and legitimize outlier ideas even when formal optimization systems discourage deviation.

Taken together, these recommendations address core challenges associated with managerial decision support and organizational sensemaking in AI-intensive environments. By embedding these design principles, organizations can capture AI-driven efficiency gains while preserving the innovative capacity necessary for sustained competitive advantage.

### 5.3 Limitations and future research directions

The propositions advanced in this article require systematic empirical testing. Table 4 provides a unified operationalization framework specifying, for each proposition, the independent and dependent variables, observable indicators, evidence types, and disconfirmation criteria—the observable evidence that would warrant rejection or substantial revision of the proposition. This framework serves as a bridge between the theoretical model and the concrete research designs outlined below.

**Table 4:** Operationalization and testing framework for propositions.

Proposition	Independent variable(s)	Dependent variable(s)	Observable indicators	Evidence types	Disconfirmation criteria	Moderating scenarios
P1a (Algorithmic tendency)	AI training objectives (optimization, pattern fitting, alignment)	Bias toward efficiency pole	Output distribution across mainstream vs. marginal inputs	AI system logs; cross-model analysis	An AI system explicitly regularized for output diversity (sacrificing some predictive accuracy for non-mainstream coverage) still exhibits exclusion patterns indistinguishable from a purely accuracy-optimized system.	Weaker in generative or exploratory AI systems; stronger in discriminative, high-stakes applications
P1b (Design conditions for bias entrenchment)	(i) Absence of outlier pathways; (ii) AI output as default (no procedural ratification)	Institutional interface bias	Proportion of low-confidence proposals filtered without review; presence of procedural approval requirements	System documentation; filtering logs; interviews	Organizations with both outlier pathways and advisory AI configuration show no statistically significant reduction in residual exclusion compared to those lacking both features.	Weaker under compliance-oriented regulation (e.g., FDA) and in-house AI development; stronger under risk-minimization regulation (e.g., AML) and vendor-locked AI
P1c (Mechanisms of entrenchment)	Conditions specified in P1b	Shift toward unconscious automatic execution	Composite score: perceived transparency, override success rate, standard review frequency	System logs + surveys + interviews; longitudinal	Under P1b conditions, none of the three dimensions (opacity, agency loss, reflexivity reduction) shows a sustained increase relative to baseline.	Same as P1b
P2a (Data feedback)	AI retraining frequency on internal data; pipeline completeness	Training data homogenization	Change in feature variance across cycles; residual proportion in pipeline	Training data snapshots; pipeline audit	After controlling for external data inputs, training data variance or entropy does not decrease over successive retraining cycles despite systematic residual exclusion.	Stronger in larger organizations with established routines; weaker in smaller, flatter organizations
P2b (Cognitive feedback with moderating conditions)	Duration of exposure to homogeneous AI outputs	Critical reflection capacity	Critical reflection scores; AI error detection in experimental tasks	Longitudinal surveys; experiments; interviews	(a) Critical reflection capacity does not diminish after prolonged homogeneous AI exposure even without mandate and cognitive slack; or (b) habituation occurs despite the presence of both moderators.	Weaker in high power distance cultures (suppress dissent); stronger where constructive challenge is encouraged
P2c (Ongoing institutional reproduction)	Marginalization or exit of heterogeneous cognition holders	Political capacity for interface adjustment	Retention of divergent profiles; frequency of reform proposals	HR records; proposal archives; exit interviews	Organizations that retain heterogeneous cognition holders show no significant difference in political capacity (e.g., reform proposal frequency) compared to high-exit organizations.	Stronger in large organizations with hierarchical structures
P2d (Crisis-driven punctuation)	External shocks (market decline, competitor disruption, algorithmic scandal)	Interface reconfiguration	Documented changes in AI parameters, protocols, or allocation criteria post-shock	Organizational archives; media; before-and-after comparison	Following a major external shock (e.g., market crash >20%), no documented interface design changes occur within 24 months.	N/A
P3a (Non-optimization zones)	Designated low-AI-involvement domains with dedicated budget and authority	Survival of trial-and-error behaviors	Exploratory activity time; projects outside KPI; innovation from protected zones	Activity logs; project records; innovation metrics	Organizations with designated non-optimization zones show no statistically significant increase in exploratory activity (e.g., out-of-KPI project time) compared to matched controls.	More effective in flatter organizational structures; may be symbolic in vendor-locked environments
P3b (Outlier pathways)	Structured human review for low-confidence outputs, with cross-functional authority and budget	Visibility of marginal cognitions	Proportion reaching review; reversal rate; panel diversity	Review records; reversal logs	(a) No documented reversals of AI judgments after human review over 12 months; or (b) human review panels reproduce AI filtering.	Weaker in high power distance cultures; stronger where constructive challenge is encouraged
P3c (Paradox-balancing roles)	Roles with cross-interface authority, ambidextrous evaluation, and discretionary budget	Plurality of value perspectives	Diversity of funded projects; minority viewpoint representation; evaluation balance	Allocation records; committee composition; evaluations	(a) No significant increase in value diversity (e.g., minority-viewpoint project funding) compared to controls; or (b) resource allocations correlate solely with efficiency metrics and not with innovation potential.	More effective with direct top-management reporting; attenuated in environments with short-tenure leadership
P4 (Secondary paradoxes)	Implementation of ambidextrous features	Emergence of secondary paradox tensions	Translation failures; leader divergence; metric or outlier-label gaming	Longitudinal case studies; interviews; behavioral metrics	After 3+ years of implementing all three ambidextrous features, the organization shows no evidence of any secondary paradox tension (isolation, authority, or evaluation) across multiple data sources.	N/A

P2d and P4 are not assigned moderating scenarios. P2d concerns exogenous crisis-driven punctuation, which by definition overrides routine boundary conditions; P4 addresses meta-level paradoxes inherent to ambidextrous design rather than context-specific moderators.

Several features of Table 4 merit emphasis. First, Proposition 1a is formulated as a conditional, testable claim: it would be disconfirmed if AI outputs exhibited no systematic preference for mainstream over marginal patterns. Second, Proposition 2 is decomposed into four independently testable sub-propositions (P2a–P2d), each with distinct variables and disconfirmation criteria. Third, indicators for the ambidextrous interface features (P3a–P3c) are specified at the organizational level, enabling comparative case designs. Fourth, residuals are operationalized using a disjunctive logic across three structural attributes. Building on this operationalization framework, this study now outlines concrete research designs capable of testing these propositions.

#### 5.3.1 Case-selection logic

For a most-similar-cases design, organizations should be matched on industry sector, size and age, and type of AI application (George and Bennett, 2005; Zuschke, 2020). The differentiating variable is the presence of ambidextrous interface features (P3a–P3c); organizations are classified as “ambidextrous” if they exhibit at least two of the three features. Five data sources are recommended: AI system logs (confidence scores, filtering records, retraining cycles), organizational documents (design specifications, review protocols), semi-structured interviews with R&D personnel and managers, archival innovation outcomes (patent filings, new product launches), and surveys measuring perceived cognitive diversity and critical reflection on AI outputs. Key validity threats include selection bias (mitigated by matching on pre-AI innovation metrics and within-case process tracing), history effects (mitigated by longitudinal tracking and same-industry control cases), researcher bias in identifying residuals (mitigated by Delphi panels with explicit reporting of evaluator backgrounds), and limited generalizability (partially offset by delineating boundary conditions and calling for replication across diverse contexts). Building on this case-selection logic, several boundary conditions merit explicit consideration as they may moderate the strength and direction of the proposed effects.

#### 5.3.2 Boundary conditions

Three boundary conditions merit explicit consideration as they may moderate the strength and direction of the proposed effects. First, industry regulation shapes the extent to which institutional interface bias materializes. Compliance-oriented mandates—such as FDA or EMA documentation requirements—function as de facto outlier pathways that weaken the bias predicted in P1b–P1c. Risk-minimization regulations—such as anti-money laundering requirements in financial services—penalize false negatives, incentivizing heavier reliance on AI outputs as default decisions and strengthening the bias (P2a–P2c). Second, AI provenance affects the transparency and contestability of interface operations. In-house development retains control over training data, confidence thresholds, and update cycles, making the opacity dimension of unconscious automatic execution more contestable and thereby weakening the mechanisms specified in P1b–P1c. Vendor-supplied systems often operate as black boxes, limiting organizational capacity to question or revise algorithmic judgments—attenuating the effectiveness of P3a–P3c. Third, organizational size and culture moderate both the emergence of interface bias and the effectiveness of ambidextrous interventions. Larger organizations tend to exhibit stronger inertial lock-in due to established routines and hierarchical structures, which may intensify the self-reinforcing dynamics of P2a–P2c. Conversely, flatter structures in smaller organizations may facilitate the implementation of ambidextrous features (P3a–P3c), though resource constraints may limit scalability (Zahoor et al., 2022). Cultural context further moderates these effects: high power distance may suppress dissent and amplify marginalization at the value interface, whereas cultures that encourage constructive challenge may weaken the bias (Van Evera, 1997).

A further boundary condition concerns AI generational evolution: generative AI's capacity for novel combination may partially counteract ghosting and squeeze effects, yet its tendency toward hallucination may prompt stricter filtering that inadvertently captures genuine innovations. Across successive AI generations, interface bias may deepen through progressive data pipeline filtration, as each generation trains on outputs that already exclude prior residuals—warranting longitudinal research across AI transitions. Organizational size, AI application type (decision support vs. process automation), and cultural trust in AI may further moderate the emergence of institutional bias and the effectiveness of mitigation strategies.

#### 5.3.3 Critical questions for future inquiry

Several questions remain central for continued scholarly exploration. Whose innovations are systematically filtered out, and how do patterns of exclusion intersect with social identity dimensions such as race, gender, and class? What happens to organizational memory when AI systems discard low-confidence ideas without archival traces—does this produce a form of organizational amnesia that undermines learning from past exclusions? Can organizations design AI systems that are paradox-aware, capable of recognizing and signaling their own filtering tendencies? Finally, what are the implications for organizational democracy when algorithms increasingly define legitimate knowledge, authority, and action?

### 5.4 A note on positionality

This article acknowledges that this theoretical framework reflects particular assumptions and exclusions. As management scholars operating within a tradition that emphasizes organizational performance and design, the focus on “innovation drivers” may privilege certain forms of novelty (e.g., product innovation) over others (e.g., social innovation). The academic positioning of this work within the Global North also shapes the questions considered legitimate and the audiences primarily addressed. Moreover, the concept of “residuals,” although intended to illuminate processes of exclusion, may inadvertently reproduce a functionalist bias by framing exclusion as a phenomenon to be optimized away rather than as a site of political contestation. Readers are therefore invited to engage critically with these limitations and to develop alternative perspectives—drawing, for example, on critical management studies, postcolonial theory, or science and technology studies—that may reveal dimensions overlooked in this analysis. This framework is not intended as a definitive conclusion but rather as an invitation to ongoing critical dialogue.

## 6 Conclusion

This article has advanced a set of context-contingent propositions suggesting that AI does not inherently erode innovation drivers, but rather creates a systematic tendency toward such erosion under specific, identifiable institutional design conditions—namely, when AI outputs serve as default decisions and outlier pathways are structurally absent. The institutional interface lens and its associated concepts—unconscious automatic execution, residuals, and the ambidextrous interface—offer analytical tools for understanding this tendency, yet the propositions remain provisional and subject to empirical validation.

The framework suggests that when AI output functions as the default decision and outlier pathways are absent, filtering mechanisms across cognitive, action, and value layers may systematically transform innovation drivers into residuals, generating a self-reinforcing tendency toward eroded organizational reflexivity. Interrupting this cycle requires deliberate institutional design choices: non-optimization zones supported by translation pathways, outlier review processes that counteract cognitive lock-in, and paradox-balancing roles with ambidextrous evaluation—while remaining attentive to the secondary paradoxes these interventions may create.

By offering a theoretically precise and empirically testable framework, this article provides analytical tools and design directions for innovation governance in AI-augmented organizations. The empirical status of these propositions, however, remains open; their validation, refinement, or falsification awaits systematic investigation across diverse organizational settings.

## Data Availability

The original contributions presented in the study are included in the article/supplementary material, further inquiries can be directed to the corresponding author.

## Footnotes

1 This vignette is a constructed composite, synthesized from patterns documented across multiple industry accounts and scholarly case studies of AI implementation in R&D contexts, rather than a description of a specific, identifiable firm. The figures cited are illustrative estimates intended to render the dynamic tangible, not empirically sourced data points. The point is not that a human expert panel possesses an infallible capacity to identify breakthroughs. Rather, the case highlights a process failure: the AI's operation rendered these ideas institutionally invisible, foreclosing any possibility of organizational deliberation about them. It is this foreclosure of the decision-making process itself that this framework seeks to theorize. Recent empirical work has documented analogous patterns: AI adoption simultaneously enhances efficiency pressures while inhibiting innovative behaviors (Wang and Long, 2025), and strategic thinking in AI-augmented teams tends to converge toward historical benchmarks, reducing cognitive diversity over time (Nyenno, 2026). These studies lend empirical plausibility to the stylized pattern described here.
